# Effects of probiotics on heart failure: a systematic review and meta-analysis

**DOI:** 10.3389/fnut.2025.1708678

**Published:** 2025-12-05

**Authors:** Zheqin Zhu, Aoli Chen, Min Wang, Huimin Zhang, Sisi Dai, Rongzhen Liu, Jianhe Liu

**Affiliations:** 1 Department of Cardiovascular Medicine, The First Affiliated Hospital of Hunan University of Chinese Medicine, Changsha, China; 2 Department of Cardiovascular Medicine, Putuo Hospital Affiliated to Shanghai University of Chinese Medicine, Shanghai, China

**Keywords:** meta-analysis, heart failure, probiotics, cardiac function, adjuvant therapy

## Abstract

**Background:**

Heart failure (HF) is a serious clinical syndrome with substantial health threats. Emerging studies link intestinal flora dysbiosis to HF onset and progression. Although probiotics are thought to regulate gut microbiota, the specific impact of probiotics on HF remains unclear, highlighting the need for systematic evaluation.

**Methods:**

As of 9 September 2025, we searched eight major academic databases using a predefined protocol for data extraction and quality assessment. Subsequently, a meta-analysis was conducted using Review Manager 5.4 and Stata 18. Forest plots were used to analyse the effect size, and publication bias was evaluated through funnel plots.

**Results:**

Ultimately, 11 of the studies met the inclusion criteria for the systematic review. The results showed that probiotics have a slight beneficial effect on cardiac function indicators (LVEF, LVESV), reduced the levels of inflammatory factors (hs-CRP, IL-6, TNF-*α*), regulated the proportion of dominant gut bacteria, and decreased the readmission rates of patients with HF. However, no beneficial effects were found on NT-proBNP, activity endurance, TMAO, and mortality.

**Conclusion:**

Probiotics exert cardioprotective effects and can serve as adjunctive therapy for HF management. Future high-quality, large-sample clinical studies are needed to further clarify their long-term efficacy and optimal intervention strategies.

**Systematic review registration:**

Details of the protocol for this systematic review were registered on PROSPERO (CRD420251083960).

## 1 Introduction

Heart failure (HF) is a complex clinical syndrome that represents the advanced stage of various heart conditions. Statistics indicate that the prevalence and mortality of HF are rising annually, affecting more than 64 million individuals globally and causing approximately 1.73 million deaths each year (1). This makes HF a significant public health concern worldwide. The pathogenesis of HF is highly complex, involving disruptions in neuro-humoral regulation, myocardial remodeling, inflammation, and oxidative stress, among other contributing factors. While substantial advancements have been made in drug therapies and device therapies in recent years, the quality of life and long-term prognosis for patients remain inadequate. Consequently, investigating the pathophysiological mechanisms underlying HF and identifying new intervention targets have become prominent research areas.

In recent years, the rapid advancements of microbiome research have gradually illuminated the link between gut microbiota (GM) and various diseases, including HF. The GM is often referred to as the “second genome” of the human body, playing a crucial role in regulating metabolism, immunity, and intestinal barrier function (2). The “Heart Failure Gut Hypothesis” posits that during HF, reduced cardiac output and systemic congestion result in insufficient intestinal perfusion, leading to intestinal ischemia, compromised barrier function, microbiota imbalance, bacterial translocation, and endotoxemia. These changes activate systemic inflammation, creating a vicious cycle that exacerbates myocardial damage and worsens cardiac function (3). This hypothesis not only offers a novel perspective on the pathogenesis of HF but also suggests that regulating the GM could emerge as a potential target for HF treatment.

Probiotics have been shown to be an effective strategy for regulating intestinal flora and enhancing the functionality of the intestinal barrier. Previous studies have demonstrated that probiotics can reduce risk factors associated with cardiovascular disease, such as blood lipid levels (4). Subsequent research further revealed the anti-inflammatory, antioxidant, and intestinal microbiota-regulating effects of probiotics under conditions of cardiac remodeling after myocardial infarction (5). This underscores the significant potential of probiotics in improving cardiac health. Numerous animal and clinical studies have investigated the effects of probiotics on HF and their underlying mechanisms, including the regulation of intestinal flora composition, reduction of harmful metabolites (such as trimethylamine-N-oxide, TMAO), promotion of beneficial metabolites (like short-chain fatty acids, SCFAs), inhibition of inflammatory signaling pathways, and alleviation of myocardial fibrosis (6–8).

While previous studies have investigated the benefits of probiotics for HF from various perspectives, the inconsistency in certain outcome measures complicates the clear definition of the role and position of probiotics in HF treatment. Therefore, we conducted a systematic review and meta-analysis of a randomized controlled trial (RCT) on the effects of probiotic supplements on patients with HF to comprehensively evaluate the role of probiotics in the management of HF.

## 2 Methods

This systematic review followed the Preferred Reporting Items for Systematic Reviews and Meta-analyses (PRISMA) guidelines and the recommendations of the Cochrane Collaboration. Details of the protocol for this systematic review were registered on PROSPERO (CRD420251083960).

### 2.1 Search strategy

Computerized searches were conducted across eight major databases: PubMed, Embase, Cochrane, Web of Science, CNKI, Wanfang Database, the United States Clinical Trial Registry, and the Chinese Clinical Trial Registry. Search the published literature from the establishment of the database until September 9, 2025. The search did not limit the publication language of the literature. The search strategy incorporated subject terms and free-text keywords, developed through iterative pre-searches. Additionally, manual searching was employed as a supplementary method. Detailed information regarding the search strategy can be found in the Supplementary File 1.

### 2.2 Eligibility criteria

Inclusion criteria: (1) Participants: Patients diagnosed with HF. (2) Intervention: Trials where the treatment group received probiotic intervention (including single-strain or multi-strain preparations), with probiotics administered as monotherapy or adjuvant therapy, and the control group received a placebo. (3) Outcome Indicators: Studies must include at least one primary or secondary outcome indicator. Primary outcome indicators include cardiac function and N-terminal pro-B-type natriuretic peptide (NT-proBNP). Secondary indicators encompass factors related to inflammation, intestinal flora abundance, activity endurance, readmission rate, and mortality. (4) Study Design: RCT.

Exclusion criteria: (1) Not an RCT; (2) The intervention measure is not probiotics; (3) Duplicated literature; (4) Reviews, comments, conference papers, dissertations; (5) Full text not available; (6) No available outcome indicators.

### 2.3 Study screening and data extraction

Two researchers independently screened the literature using NoteExpress (version 4.2.0.10156) software based on the inclusion and exclusion criteria. Translation tools were used to assist in determining eligibility for inclusion and reducing single-language comprehension biases. In instances of disagreement between the two researchers, they consulted a third researcher to reach a consensus. The extracted data included authors’ names, publication year, country or region, the sample sizes of the experimental and control groups, age, sex ratio, specific intervention measures, intervention duration, and outcome indicators. When results were only available in graphical format, the GetData2.26 software extracted numerical values from the graphs. This software has been effectively utilized in other meta-analyses and is recognized as a reliable method for extracting data from studies (9).

### 2.4 Risk of bias

The Cochrane Collaboration risk of bias tool was used to evaluate the quality of the literature in seven aspects: random sequence generation, allocation concealment, blinding of participants and personnel, blinding of outcome assessment, incomplete outcome data, selective reporting, and other bias. Each aspect was categorized as “low risk” “unclear risk” or “high risk.” Two researchers conducted independent evaluations, and in instances of disagreement, they consulted with a third researcher to achieve consensus.

### 2.5 Statistical analyses

Meta-analysis was conducted utilizing Review Manager 5.4 and Stata 18. The results for binary variables were expressed as relative risk (RR) along with a 95% confidence interval (95% CI), while continuous variables were presented as either mean difference (MD) or standardized mean difference (SMD). The heterogeneity among the studies was assessed using *I*^2^ statistics and *p*-values from the chi-square test. When *I*^2^ < 50% and *p* > 0.1, it indicated no heterogeneity among the studies, and a fixed-effect model was used; otherwise, a random-effect model was adopted. Sensitivity analysis or subgroup analysis was performed on outcomes with high heterogeneity to identify the sources of heterogeneity, and the stability and reliability of the overall results were evaluated simultaneously. Funnel plots were generated using Stata 18 to test and assess publication bias. Additionally, the Kappa value was calculated using SPSS 25.0 software to assess the degree of consistency between the two researchers’ literature screening and quality evaluation results. The results are presented in Supplementary File 1.

## 3 Result

### 3.1 Results of study search and selection

**Figure 1:**
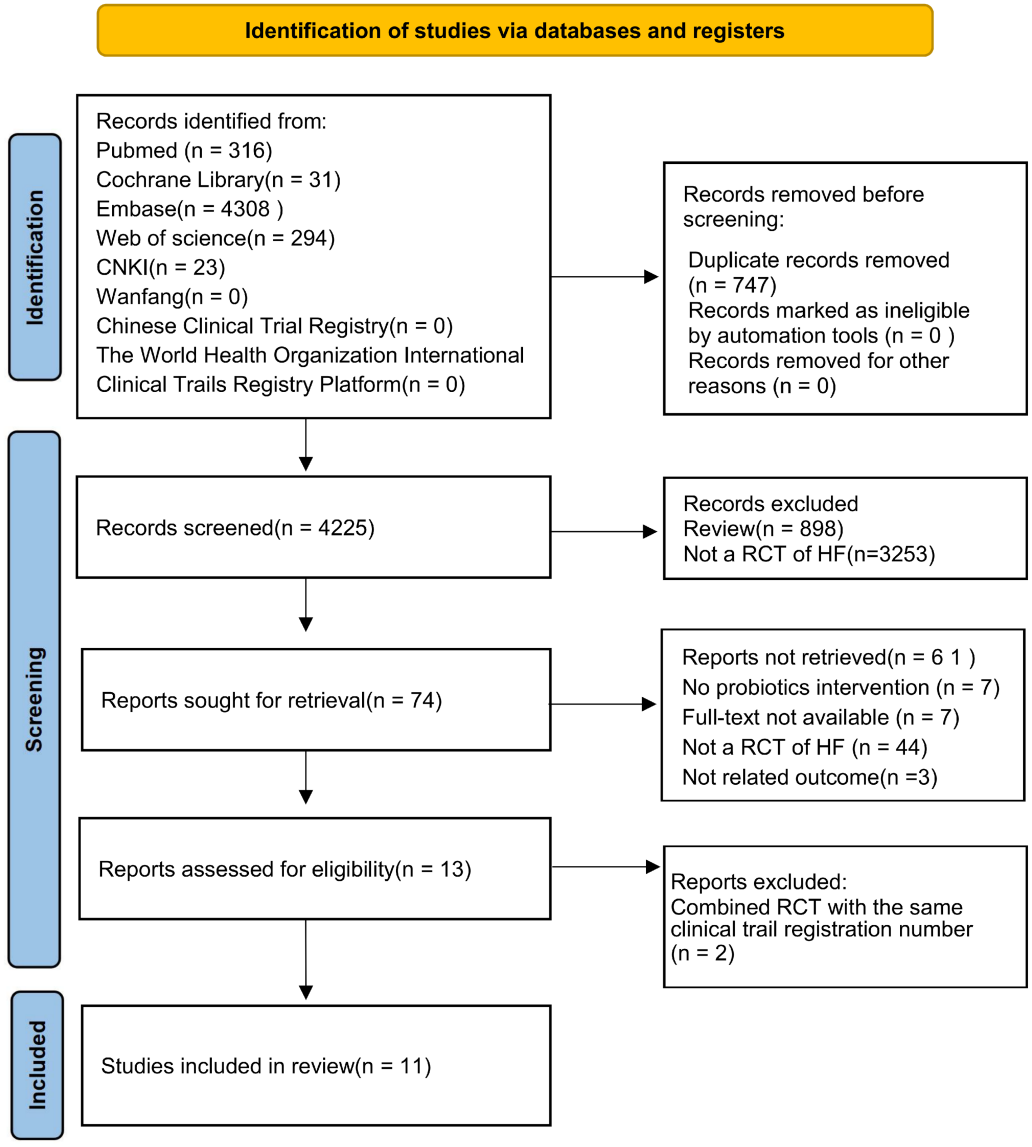
Flow chart of the process of study selection.

The preliminary search yielded 4,972 articles that met the established criteria. After eliminating 747 duplicate articles using NoteExpress and screening for relevant research content by reviewing titles and abstracts, 74 articles were obtained. After excluding articles where the RCTs were not related to HF, those with intervention measures other than probiotics, those without full-text access, and those without available outcome indicators, 13 articles were selected for further review. After merging the literature with the same clinical trial registration number, a total of 11 studies were ultimately included in meta-analysis. The detailed literature screening process is shown in Figure 1.

### 3.2 Characteristics of included studies

Table 1 shows the main characteristics of the included clinical studies. A total of 11 RCTs were included (8, 10–21), involving 827 participants. Six of the studies were conducted in China (8, 11, 13, 14, 19, 21), two in Iran (12, 16, 17, 20), and one each in Brazil (10), Norway (15), and the United Arab Emirates (18). Among the included studies, 10 studies (8, 10, 12–16, 19, 21) specified the New York Heart Association’s cardiac function classification for the participants, and four studies included patients with heart failure with reduced ejection fraction (HFrEF) (12, 15, 16, 18). The average treatment duration was 11.5 weeks. The most commonly used probiotic genera were *Lactobacillus* and *Bifidobacterium*.

**Table 1:** Characteristics of clinical studies (*n* = 11).

Study (year)	Country	Participants (Experimental/Control group)	Male/ Female	Age (mean±SD)	Types of heart failure	Experimental group	Control group	Time of treatment	Outcomes	Rehospitalization rate and mortality rate
Costanza AC (2015) (10)	Brazil	8/8	NR	NR	NYHA II-III	*Saccharomyces boulardii*, 1,000 mg/day	Placebo	12 weeks	hsCRP LVEF	NR
Yu H (2021) (11)	China	5/5	C:3/2 E:2/3	C:70.14 ± 4.71 E:70.21 ± 4.78	NR	*Bifidobacterium longum*, *Lactobacillus bulgaricus* and *Streptococcus thermophilus*, 1.2 × 10^7^ CFU/g, 2 g × 3 times/day	Dietary management	6 weeks	hsCRP TNF-α IL-6 Abundance of intestinal flora	NR
Matin SS (2021,2024) (12, 17)	Iran	41/39	C:25/14 E:32/9	C:50.1 ± 10.56 E:50.54 ± 11.4	NYHA I-III, HFrEF	*Lactobacillus casei* (10^9^CFU), *Lactobacillus acidophilus* (1.5 × 10^10^ CFU), *Lactobacillus rhamnosus* (3.5 × 10^9^ CFU), *Lactobacillus bulgaricus* (2.5 × 10^8^ CFU), *Bifidobacterium breve* (10^10^ CFU), *Bifidobacterium longum* (5 × 10^8^ CFU), *Streptotus thermophilus* (1.5 × 10^8^ CFU)(0.5 g/day)	Placebo	10 weeks	sCD163 sTWEAK physical activity NTpro-BNP hs-CRP	Experimental/Control group: The number of deaths: 1/1.
Ding J (2021) (8)	China	50/50	C:29/21 E:27/23	C:65.86 ± 5.35 E:67.96 ± 5.96	NYHA II-III	*Bifidobacterium longum*, *Lactobacillus bulgaricus* and *Streptococcus thermophilus*, 1.2 × 10^7^ CFU/g, 2 g × 3 times/day	Conventional treatment	12 weeks	LVEF TNF-α hsCRP TMAO	NR
Li YL (2024) (13)	China	56/56	C:29/27 E:30/26	C:63.15 ± 4. 82 E:62.45 ± 5.12	NYHA II-III	*Bifidobacterium longum*, *Lactobacillus acidophilus* and *Enterococcus faecalis*, 10^7^CFU/0.21 g，0.42 g × 3 times/day	ARB + ACEI	16 weeks	LVEF LVESV Abundance of intestinal flora TMAO TNF-α	Experimental/Control group: ① The number of deaths: 1/4. ② The number of readmission patients: 7/17.
Wang J (2023) (14)	China	43/43	C:26/17 HF:25/18	C:67.23 ± 7.36 HF:68.02 ± 7.43	NYHA II-IV	*Bifidobacterium infantis*, *Lactobacillus acidophilus* and *Enterococcus faecalis* (0.5 × 10^6^ CFU), *Bacillus cereus* (0.5 × 10^5^ CFU), 1.5 g × 3 times/day	Conventional treatment	12 weeks	LVEF LVESV hs-CRP TNF-α IL-6	NR
Ayodeji A (2021) (15)	Norway	52/51	C:38/13 E:42/10	C:60 ± 10 E:59 ± 10	NYHA II-III, HFrEF	*Saccharomyces boulardii*, (7.5 × 10^9^ CFU/250 mg, 500 mg × twice/day)	Conventional treatment	12 weeks	LVEF NTpro-BNP CRP TMAO	Experimental/Control group: The number of readmission patients: 2/3.
Pourrajab B (2020,2022) (16, 20)	Iran	39/39	C:26/13 E:29/10	C:53.87 ± 7.25 E:55.59 ± 8.95	NYHA I-III，HFrEF	*Lactobacillus acidophilus La5* + *Bifidobacterium lactis Bb12*, 3 × 10^9^ CFU,300 mL/day	Ordinary yogurt	10 weeks	NTpro-BNP physical activity sCD163 sTWEAK	NR
Karim A (2022) (18)	United Arab Emirates	44/48	C:48/0 E:44/0	C:65.2 ± 5.6 E:67.6 ± 4.9	HFrEF	*Bifidobacteria+lactobacilli*+*Streptococcus thermophilus*, 1.1 × 10^11^ CFU/0.676 g, 0.676 g/day	Placebo	12 weeks	LVEF CRP	NR
Chen LN (2023) (19)	China	50/50	C:26/24 E:22/28	C:70.60 ± 6.24 E:73.16 ± 8.33	NYHA II-III	*Bifidobacterium longum*, *Lactobacillus acidophilus* and *Enterococcus faecalis*, 10^7^CFU-0.21 g, 0.63 g × twice/day	Conventional treatment	12 weeks	LVEF NTpro-BNP TMAO	Experimental/Control group: ① The number of deaths: 1/0. ② The number of readmission patients: 6/14.
Zhang Y (2017) (21)	China	25/25	C:23/2 E:23/2	C:86.2 ± 4.6 E:85.9 ± 4.3	NYHA II-III	*Bifidobacterium longum*, *Lactobacillus acidophilus* and *Enterococcus faecalis*, 10^7^CFU-0.21 g，0.42 g × 3 times/day	Conventional treatment	12 weeks	LVEF	NR

C, Control group; E, Experimental group; NYHA, New York Heart Association; HFrEF, Heart failure with reduced ejection fraction; hsCRP, Hypersensitive C-reactive protein; NR, Not report; LVEF, Left ventricular ejection fraction; TNF-α, Tumor necrosis factor-α; IL-6, Interleukin- 6; sCD163, Soluble CD163; sTWEAK, soluble tumor necrosis factor-like weak inducer of apoptosis; NTpro-BNP, N-terminal pro-brain natriuretic peptide; TMAO, Trimethylamine N-oxide; ARB, Angiotensin II Receptor Blockers; ACEI, angiotension converting enzyme inhibitors; LVESV, Left ventricular end systolic volume.

### 3.3 Study quality

**Figure 2:**
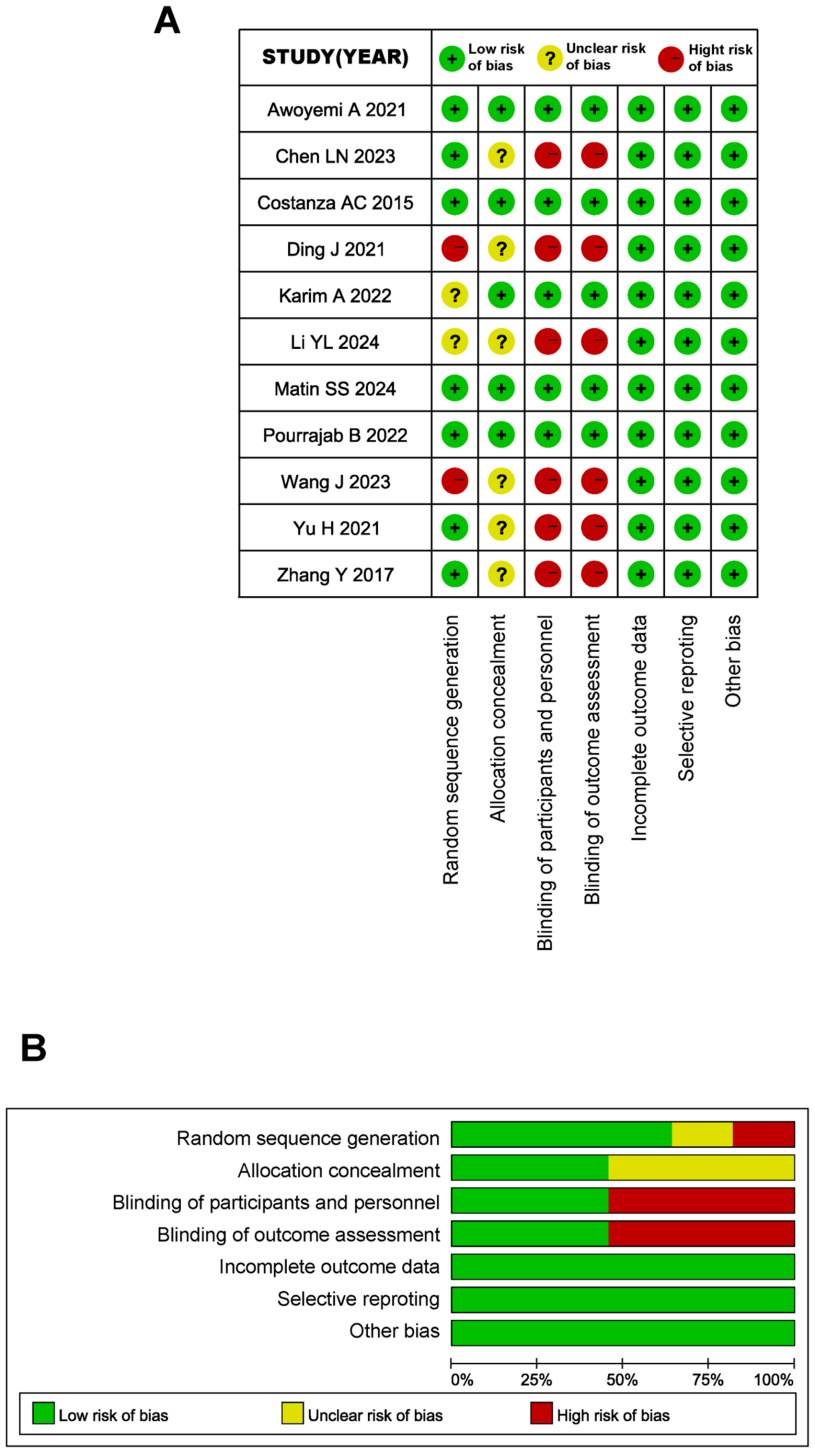
Quality assessment of RCTs. **(A)** Risk of bias summary of RCTs. **(B)** Risk of bias graph of RCTs.

Figure 2 shows the assessment of study quality and risk of bias, using the Cochrane Collaboration risk of bias tool. The studies by Awoyemi A (15), Costanza AC (10), Matin SS (12), and Pourrajab B (16) indicated a lower risk of bias. The studies by Ding J (8), Li YL (13), and Wang J (14) indicated a higher risk of bias. In general, the blinding of participants, researchers, and outcome assessors, the generation of random sequences, and the allocation concealment process are aspects with a higher risk of bias.

### 3.4 Meta-analysis results

#### 3.4.1 Effects of probiotics on cardiac function

**Figure 3:**
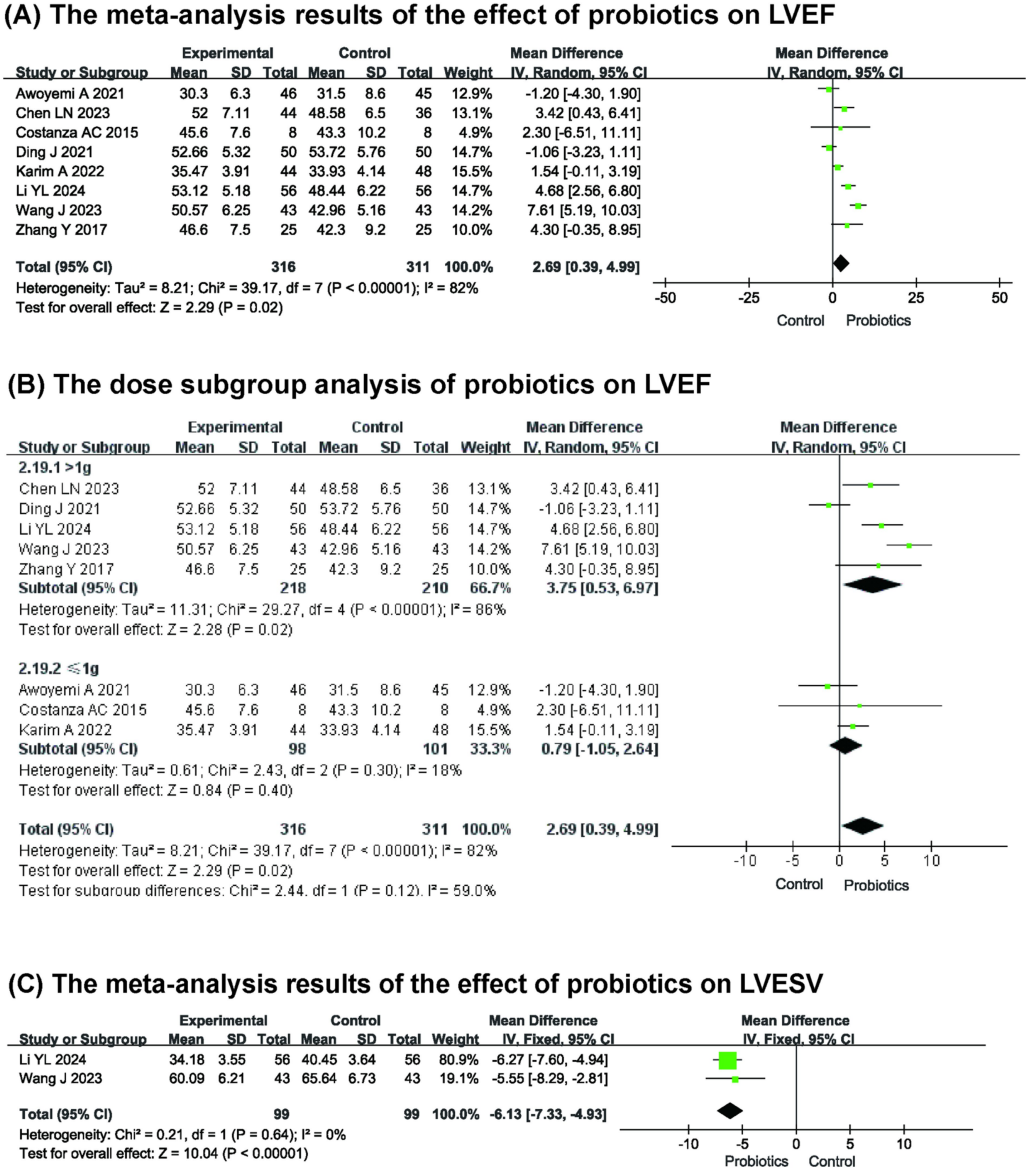
**(A)** The meta-analysis results of the effect of probiotics on LVEF **(B)** The dose subgroup analysis of probiotics on LVEF **(C)** The meta-analysis results of the effect of probiotics on LVESV.

Eight studies (8, 10, 13–15, 18, 19, 21) reported left ventricular ejection fraction (LVEF). Figure 3A shows the meta-analysis results: compared with the control group, probiotics increased LVEF (*n* = 627, MD: 2.69, 95% CI (0.39, 4.99), *p* = 0.02, *I*^2^ = 82%). Due to the significant heterogeneity observed among the included studies, a sensitivity analysis was performed. Although we sequentially excluded each study, the source of the heterogeneity remained unclear. Nevertheless, the sensitivity analysis indicated that the results were stable.

We further explored the source of heterogeneity by dividing the subjects into two subgroups based on probiotic dose (> 1 g and ≤ 1 g) for subgroup analysis. The results are shown in Figure 3B. When the probiotic dose was ≤ 1 g, probiotics had no significant effect on LVEF compared to the control group (MD: 0.79, 95% CI (−1.05, 2.64), *p* = 0.40), and the heterogeneity was low (*p* = 0.30, *I*^2^ = 18%). When the probiotic dose was > 1 g, a significant improvement in LVEF was noted (MD: 3.75, 95% CI (0.53, 6.97), *p* = 0.02), although this subgroup exhibited high heterogeneity (*p* < 0.00001, *I*^2^ = 86%). This indicates that the difference in probiotic dose may be the source of heterogeneity, and it is a relevant factor affecting the improvement effect of LVEF.

Furthermore, two studies (13, 14) reported on left ventricular end systolic volume (LVESV) (Figure 3C), revealing that probiotics significantly decreased LVESV compared to the control group (*n* = 198, MD: −6.13, 95% CI: (−7.33, −4.93), *p* < 0.00001, *I*^2^ = 0%).

#### 3.4.2 Effect of probiotics on NT-proBNP

**Figure 4:**
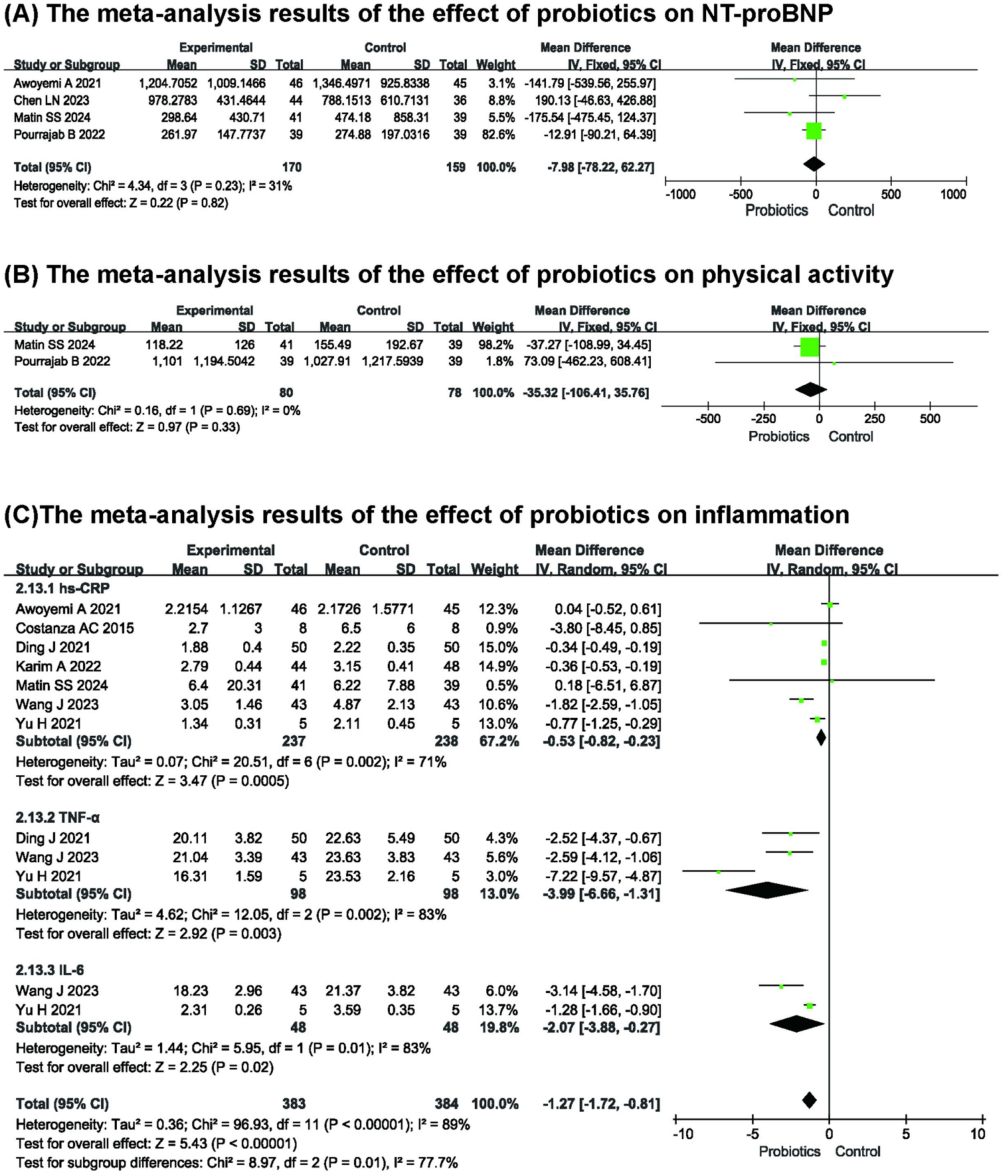
The meta-analysis results of the effect of probiotics on **(A)** NT-proBNP **(B)** physical activity **(C)** inflammation.

Four studies (15–17, 19) reported the effect of probiotics on serum NT-proBNP. The results indicated that probiotics did not reduce NT-proBNP compared with the control group (*n* = 329, MD: −7.98, 95% CI (−78.22, 62.27), *p* = 0.82, *I*^2^ = 31%) (Figure 4A).

#### 3.4.3 Effect of probiotics on physical activity

Two studies (17, 20) reported the effects of probiotics on physical activity. The meta-analysis results indicated that probiotics did not improve the activity endurance of patients with HF (*n* = 158, MD: −35.32, 95% CI (−106.41, 35.76), *p* = 0.33, *I*^2^ = 0%) (Figure 4B).

#### 3.4.4 Effect of probiotics on inflammation

A total of seven studies (8, 10, 11, 14, 15, 17, 18) examined the effects of probiotics on inflammation. The meta-analysis of hypersensitive C-reactive protein (hs-CRP), interleukin-6 (IL-6) and tumor necrosis factor-*α* (TNF-α) indicated a significant overall effect, indicating that probiotics can inhibit the systemic inflammation in patients with HF (*n* = 475, MD: −1.27, 95% CI (−1.72, −0.81), *p* < 0.00001, *I*^2^ = 89%) (Figure 4C). Regarding sensitivity analysis, the results showed no significant change in the overall effect compared with that of the subgroups.

#### 3.4.5 Effect of probiotics on the intestinal flora and its products

**Figure 5:**
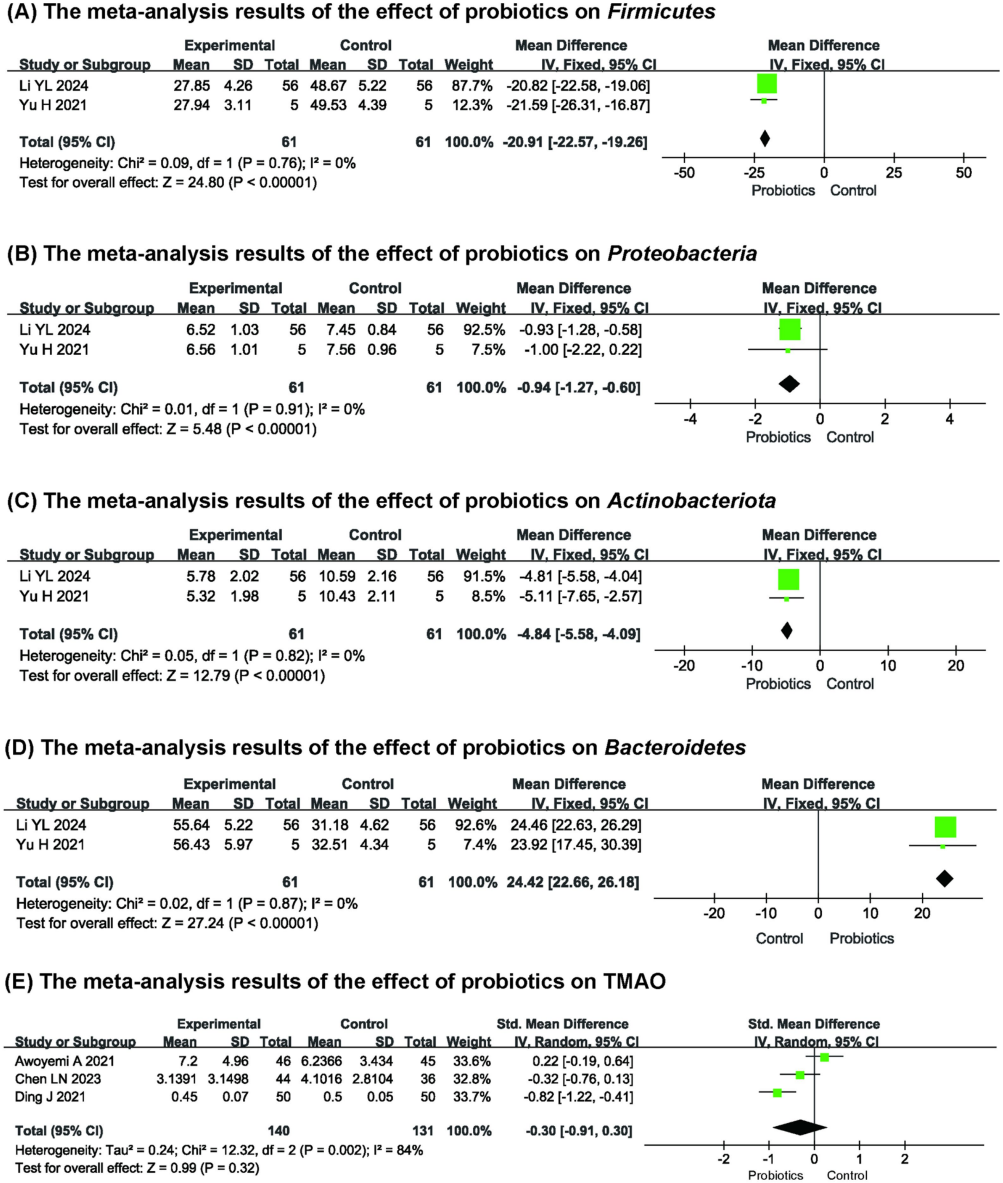
The meta-analysis results of the effect of probiotics on **(A)** *Firmicutes* **(B)** *Proteobacteria* **(C)** *Actinobacteriota* **(D)** *Bacteroidetes* **(E)** TMAO.

Two studies (*n* = 122) (11, 13) reported the effects of probiotics on the GM abundance. Among them, the *Firmicutes* and the *Bacteroidetes* were the dominant bacterial groups in the intestines of patients with HF. The results revealed that compared with the control group, probiotics could reduce the proportions of *Firmicutes* (MD: −20.91, 95% CI (−22.57, −19.26), *p* < 0.00001, *I*^2^ = 0%), *Proteobacteria* (MD: −0.94, 95% CI (−1.27, −0.60), *p* < 0.00001, *I*^2^ = 0%), and *Actinobacteriota* (MD: −4.84, 95% CI (−5.58, −4.09), *p* < 0.00001, *I*^2^ = 0%) in the intestines, and increase the proportion of *Bacteroidetes* (MD: 24.42, 95% CI (22.66, 26.18), *p* < 0.00001, *I*^2^ = 0%) (Figures 5A**–**D), thereby contributing to the regulation of GM.

Three studies (8, 15, 19) indicated that TMAO was not significantly influenced by probiotics when compared to the control group (MD: −0.30, 95% CI (−0.91, 0.30), *p* = 0.32, *I*^2^ = 84%) (Figure 5E). This finding also suggested considerable heterogeneity among the studies. In an effort to identify the source of this heterogeneity, we systematically removed each study one by one; however, the underlying cause remained elusive. Nevertheless, the sensitivity analysis demonstrated that the results were stable.

#### 3.4.6 Effect of probiotics on the readmission rate and mortality

**Figure 6:**
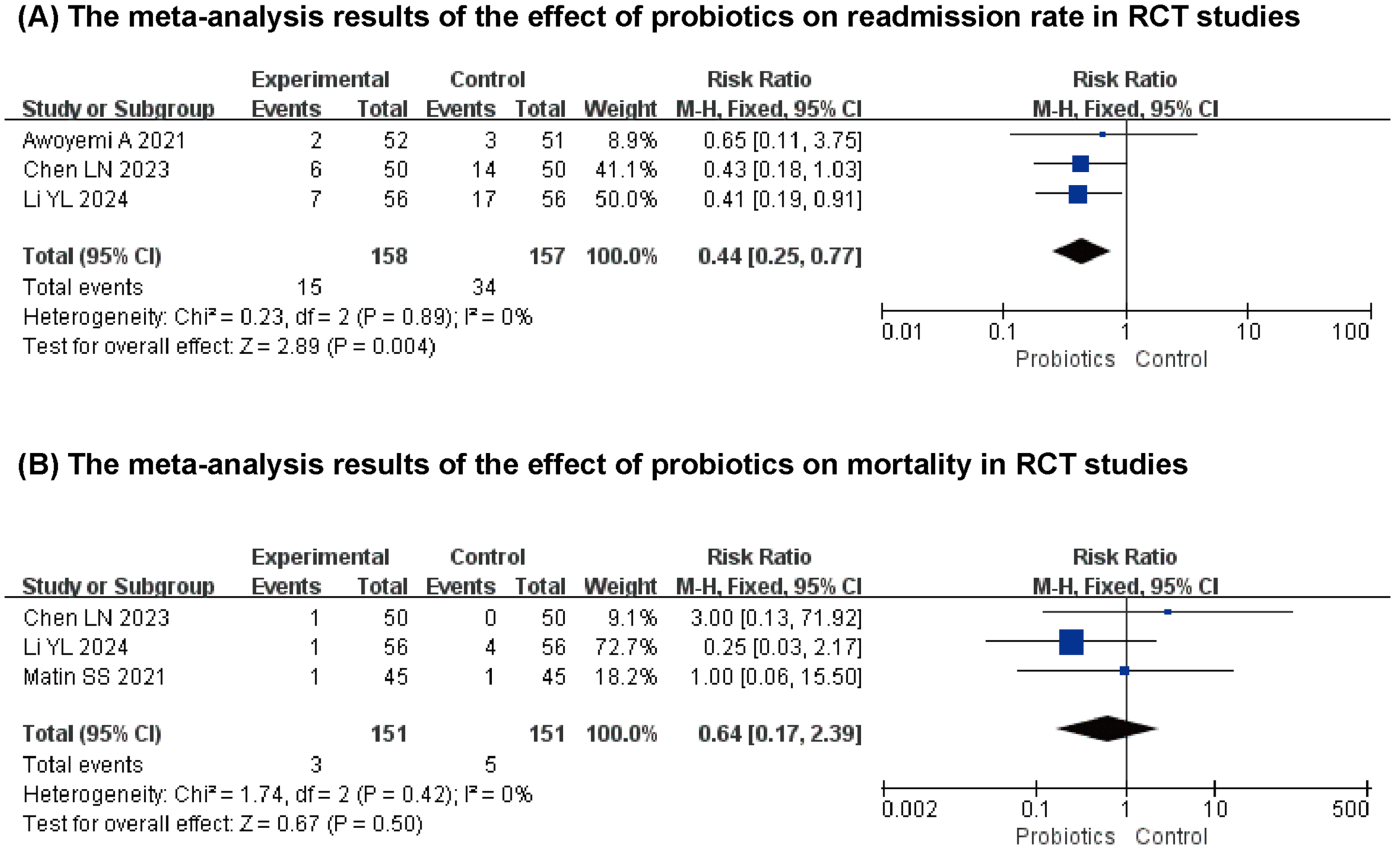
The meta-analysis results of the effect of probiotics on **(A)** readmission rate **(B)** mortality.

Three studies (13, 15, 19) reported the impact of probiotics on readmission rates. The results from the meta-analysis indicated that probiotics significantly reduced the readmission rates of patients with HF compared to the control group (*n* = 315, RR: 0.44, 95% CI (0.25,0.77), *p* = 0.004, *I*^2^ = 0%). However, a meta-analysis of the three clinical studies that evaluated mortality (13, 17, 19) revealed no significant effect of probiotics on mortality (*n* = 302, RR: 0.64, 95% CI (0.17, 2.39), *p* = 0.50, *I*^2^ = 0%) (Figure 6).

### 3.5 Publication bias

We used funnel plots to assess publication bias in the meta-analysis results with a total of ≥ 5 included studies. The funnel plots were all approximately symmetrical (Supplementary Figure S1).

## 4 Discussion

Our study included 11 RCTs involving 827 participants, aiming to review and analyse the effects of probiotic supplements on the cardiac function, overall condition, and prognosis of patients with HF, to reveal their potential benefits as supplementary treatments for HF. Previous similar studies showed that probiotics had no improvement effect on the LVEF of patients with HF (22). After including new studies, we found that probiotics could increase LVEF by 2.69%, and this improvement was manifested when the daily dose was > 1 g. However, from a clinical perspective, a 2.69% increase was within the range of routine measurement errors for LVEF. Additionally, the results indicated that probiotics had no beneficial effects on NT-proBNP or activity endurance. Nevertheless, probiotics significantly reduced the LVESV in HF patients. This suggests that probiotics have a slight improvement effect on cardiac remodeling at the microscopic level, but the efficacy is negligible for the overall complex physiological and pathological state of patients with HF.

The excessive proliferation of cardiac fibroblasts and the accumulation of extracellular matrix lead to cardiac remodeling, an important pathological precursor of HF. The cross-linked carboxyl terminal telopeptide (ICTP) is a crucial biomarker for monitoring extracellular matrix metabolism in the myocardium (23). Elevated levels of ICTP are strongly correlated with the development of HF (24). The research by Li et al. demonstrated that probiotics enhanced the inhibitory effect of the angiotensin-converting enzyme inhibitors and angiotensin II receptor blockers on the renin-angiotensin system, reduced ICTP in patients with HF, alleviated myocardial fibrosis, and thereby reversed ventricular remodeling (13). The mechanism by which probiotics inhibit myocardial fibrosis and alleviate ventricular remodeling may be related to the inhibition of inflammation, regulation of the intestinal flora, and improvement of cardiac energy metabolism.

Activation of the transforming growth factor-*β* (TGF-β) pathway is crucial for myofibroblast formation and induction, and TGF-β antibodies alleviate cardiac fibrosis and diastolic dysfunction (25). SCFAs like acetate and butyrate are metabolic products of the GM that help prevent systemic inflammation and maintain intestinal barrier integrity. An imbalance in GM can disrupt SCFA production and weaken the gastrointestinal epithelium, allowing luminal toxins to enter, triggering inflammation in the cardiovascular system and contributing to cardiac fibrosis (26). Studies indicate that HF is associated with decreased bacterial richness and a loss of butyric acid-producing bacteria (27). Wang et al. induced myocardial fibrosis in mice and found that butyric acid supplementation reduced Collagen I and TGF-β expression, preventing fibrosis progression. Additionally, *Bifidobacterium pseudolongum* enhanced butyric acid production by *Lactobacillus butyricum*, and their combined use showed a stronger anti-myocardial fibrosis effect (6). In HF, increased fatty acid oxidation inhibits glucose utilization, disrupts the glucose-fatty acid cycle in the heart, creates energy imbalance, and promotes cell damage and fibrosis activation (28). Tuerhongjiang G et al. discovered a negative correlation between intestinal *Clostridium* and circulating saturated fatty acids (SFA) in both HF patients and animal models. Administration of *Clostridium sporogenes* reduced SFA, regulated cardiac energy metabolism, and inhibited myocardial fibrosis, thereby improving cardiac function in HF mice (29).

Growing evidence shows that inflammation-related GM imbalance is linked to HF. The “intestinal leak” hypothesis suggests that GM imbalance disrupts tight junctions in intestinal cells, allowing toxins to enter the bloodstream and leading to systemic inflammation that impacts the cardiovascular system (30). Common mechanisms of intestinal damage in HF patients include reduced cardiac output, excessive activation of the sympathetic system, ischemic swelling of the intestinal mucosa, and deterioration of intestinal wall barrier function (31). Under such pathological conditions, inflammatory factors such as hs-CRP, IL-6, and TNF-*α* are produced.

Hs-CRP is a classic inflammatory marker for HF assessment and can exacerbate the disease by increasing oxidative stress and disrupting energy metabolism (32). IL-6 plays a crucial regulatory role in the inflammation associated with HF. In this context, cardiomyocytes and macrophages increase IL-6 secretion, activating NF-κB and other pathways, which recruit more inflammatory factors and amplify systemic inflammation (33, 34). Furthermore, IL-6 can promote myocardial fibrosis by activating the TGF-β1 pathway or interfering with myocardial metabolism, worsening the overall condition of HF patients through multiple mechanisms (35, 36). TNF-*α* is an important pro-inflammatory factor in the development of HF. Besides amplifying the inflammation and stimulating myocardial fibrosis, TNF-α can directly inhibit myocardial contraction by reducing sarcoplasmic reticulum proteins and impairing the efficiency of calcium uptake and release in cardiomyocytes (37). Studies have indicated that alterations in the dominant microbial population and intestinal epithelial damage in HF patients may induce the secretion of inflammatory mediators, including hs-CRP, IL-6, and TNF-α (32, 38). Our findings demonstrate that probiotic administration reduced levels of hs-CRP (8, 10, 11, 14, 15, 17, 18), IL-6 (11, 14), and TNF-α (8, 11, 14) in HF patients, with a significant overall effect. Additionally, Wang et al. conducted animal experiments that demonstrated probiotics inhibit the maturation and secretion of TNF-α and IL-6, mediated by the NOD-like receptor thermal protein domain-associated protein 3, thus regulating inflammation in HF (39).

The predominant bacterial species in the human intestinal microbiota mainly include *Firmicutes*, *Bacteroidetes*, *Proteobacteria*, and *Actinobacteria* (40). Recent studies show decreased diversity or imbalanced composition of GM in HF patients (41). Many bacterial species in the *Firmicutes* ferment dietary fibers to produce SCFAs, which can inhibit inflammation by activating G protein-coupled receptors and reducing pro-inflammatory factors (42). Studies have shown a significant increase in *Firmicutes* levels in the intestines of HF animals (43, 44). Our results show that the intervention of probiotics can reduce the proportion of the *Firmicutes* in HF patients (11, 13). This phenomenon may be caused by intestinal microcirculation disorders and abnormal permeability during HF, which trigger cytokine production and create a favorable environment for *Firmicutes* proliferation, leading to increased abundance of this phylum. Probiotics use inhibits inflammation and promotes intestinal mucosal repair, thereby alleviating the stimuli that drive *Firmicutes* proliferation. *Bacteroidetes* also contribute to SCFA production, helping maintain intestinal integrity, regulate immune function, and alleviate cardiac hypertrophy and myocardial fibrosis (45). *Proteobacteria* are a marker of GM imbalance. All members of this phylum are Gram-negative bacteria, with their outer membrane primarily composed of lipopolysaccharides (LPS). When LPS enters the bloodstream, it activates the NF-κB pathway, increasing TNF-*α*, IL-6, and IL-1β levels, which worsen systemic inflammation and HF progression (46). A meta-analysis found that in HF patients, *Bacteroidetes* decreased and *Proteobacteria* increased compared to non-HF patients (47). In light of this shift in bacterial community proportions, our findings indicated that administering probiotics increased *Bacteroidetes* and decreased *Proteobacteria* (11, 13). *Bifidobacteria* are essential members of the *Actinobacteria*, helping to reduce ammonia in feces and lower intestinal pH, creating an optimal gut environment. They also inhibit harmful bacteria and inflammatory factors (48–50). Studies indicate that *Actinobacteria*, particularly *Bifidobacteria*, increase in the intestines of HF patients, potentially due to compensatory adaptations to the intestinal environment (47, 51). The studies by Yu (11) and Li (13) demonstrated that probiotics can reduce the level of the *Actinobacteria* in the intestines of patients with HF, but the mechanisms need further investigation.

TMAO is a harmful metabolite created by gut bacteria from choline and carnitine, leading to oxidative stress and inflammation. This can result in endothelial and mitochondrial dysfunction, contributing to myocardial hypertrophy, fibrosis, and impaired cardiac function. Elevated TMAO levels are closely associated with late diastolic dysfunction of the left ventricle and affect the prognosis of patients with HF (52). Nevertheless, our meta-analysis did not find any evidence that probiotics reduce TMAO levels in patients with HF (8, 15, 19). This is related to the specificity of TMAO metabolism by the microbiota, and the probiotics we included in the study were not effective strains for reducing TMAO levels (53).

Regarding safety and prognosis, previous studies evaluated the effect of probiotics on renal function in patients with HF, and the results showed no difference compared to the control group. Our study focused on mortality and readmission rates, and the results revealed that probiotics could not reduce the mortality rate of patients with HF, but could lower the readmission rate of HF patients. This result provides a new option for improving patient prognosis and reducing medical costs while supporting the clinical application of the “gut-heart axis” theory.

Overall, probiotics act as an auxiliary regulator rather than a core treatment in HF management. They have a certain delaying effect on the structural progression of HF and have potential value in maintaining long-term cardiac function. Moreover, the anti-inflammatory and GM-regulating effects of probiotics are more targeted for patients with infections, diarrhea, and constipation, and can serve as an auxiliary measure to reduce medical expenses and improve quality of life. This conclusion encourages clinicians to apply probiotics more rationally, integrating them into the “multi-target comprehensive management strategy” for HF rather than as an independent treatment option.

## 5 Limitations

Our research has certain limitations. Firstly, the quality of the included studies was relatively low, some studies neglected the importance of randomization, blinding, and allocation concealment.

The limited number of included studies and small sample size led to three main issues. Firstly, this resulted in an overestimation of the effect size; while probiotics showed a cardioprotective effect on HF, the actual therapeutic effect was small. Combined with the insufficient sample size, this further reduced the statistical persuasiveness of the results. Secondly, the limited sample restricted deeper analysis of heterogeneity; despite conducting subgroup and sensitivity analyses, it remained challenging to clarify the real impact of potential moderating factors on the intervention effect. Lastly, Egger’s test for publication bias could not be used, making the assessment of bias rely on subjective judgment from the funnel plot, which further increased the uncertainty in assessing publication bias. Therefore, future high-quality, multicenter, and large-sample studies are needed.

Current evidence on probiotics for HF has two main shortcomings: First, there is limited exploration of underlying mechanisms; most studies focus on therapeutic indicators without identifying specific cardioprotective targets. Second, the specificity of strains is often unclear, as many studies use multi-strain combinations rather than single strains, making it difficult to assess their independent effects. Future research should examine the efficacy and dose-effect relationships of individual strains to provide clearer evidence for clinical applications.

## 6 Conclusion

In conclusion, the systematic review and meta-analysis indicate that probiotics have a protective effect on the heart and can reduce readmission rates. A daily dose of > 1 g is recommended. However, probiotics have no benefits for NT-proBNP, exercise endurance, or mortality. They can be used as an adjunctive treatment in the management of HF. Based on the evidence from RCTs, the most commonly used probiotic species are *Lactobacillus* and *Bifidobacterium*. The mechanism by which probiotics exert their effects may be related to anti-myocardial fibrosis, anti-inflammation, regulation of the GM, and improvement of myocardial energy metabolism. However, the heterogeneity of the meta-analysis results and the varying quality of some studies have diminished the credibility of the findings. Therefore, further high-quality research is needed to validate these results in the future.

## Data Availability

The original contributions presented in the study are included in the article/Supplementary material, further inquiries can be directed to the corresponding author.

## References

[1] Savarese G Becher PM Lund LH Seferovic P Rosano GMC Coats AJS. Global burden of heart failure: a comprehensive and updated review of epidemiology. Cardiovasc Res. (2023) 118:3272–87. doi: 10.1093/cvr/cvac013, 35150240

[2] Zhao L. Genomics: the tale of our other genome. Nature. (2010) 465:879–80. doi: 10.1038/465879a, 20559375

[3] Matacchione G Piacenza F Pimpini L Rosati Y Marcozzi S. The role of the gut microbiota in the onset and progression of heart failure: insights into epigenetic mechanisms and aging. Clin Epigenetics. (2024) 16:175. doi: 10.1186/s13148-024-01786-9, 39614396 PMC11607950

[4] Sun J Buys N. Effects of probiotics consumption on lowering lipids and CVD risk factors: a systematic review and meta-analysis of randomized controlled trials. Ann Med. (2015) 47:430–40. doi: 10.3109/07853890.2015.1071872, 26340330

[5] Taslim NA Yusuf M Ambari AM del Rosario Puling IM Ibrahim FZ Hardinsyah H . Anti-inflammatory, antioxidant, metabolic and gut microbiota modulation activities of probiotic in cardiac remodeling condition: evidence from systematic study and Meta-analysis of randomized controlled trials. Probiotics Antimicrob Proteins. (2023) 15:1049–61. doi: 10.1007/s12602-023-10105-2, 37349622 PMC10393865

[6] Wang J Chen J Li L Zhang H Pang D Ouyang H . Clostridium butyricum and *Bifidobacterium pseudolongum* attenuate the development of cardiac fibrosis in mice. Microbiol Spectr. (2022) 10:e0252422. doi: 10.1128/spectrum.02524-22, 36318049 PMC9769846

[7] Hesari Z Kafshdoozan K Barati M Kokhaei P Andalib S TalebiKiassari F . *Lactobacillus paracasei* impact on myocardial hypertrophy in rats with heart failure. J Chem Health Risks. (2020) 10:67–74. doi: 10.22034/jchr.2020.1888659.1080

[8] Ding J Jing HZ Wang ZL Yang Q. A study on the impact of probiotics on patients with chronic heart failure based on the analysis of the intestinal flora. J Hainan Med Univ. (2021) 27:260–4. doi: 10.13210/j.cnki.jhmu.20200902.003

[9] Khaliq MA Alsudays IM Alhaithloul H Alhaithloul HAS Rizwan M Yong JWH . Biochar impacts on carbon dioxide, methane emission, and cadmium accumulation in rice from cd-contaminated soils; a meta-analysis. Ecotoxicol Environ Saf. (2024) 274:116204. doi: 10.1016/j.ecoenv.2024.116204, 38489905

[10] Costanza AC Moscavitch SD Faria Neto HC Mesquita ET. Probiotic therapy with *Saccharomyces boulardii* for heart failure patients: a randomized, double-blind, placebo-controlled pilot trial. Int J Cardiol. (2015) 179:348–50. doi: 10.1016/j.ijcard.2014.11.034 25464484

[11] Yu H Dong AQ Zhao Y Li P Zhang Q Lu J . Study on the changes of gut microbiota in patients with coronary heart disease complicated with heart failure and the effect of probiotic intervention. Mil Med. (2021) 45:443–8.

[12] Matin SS Shidfar F Naderi N Amin A Hosseini-Baharanchi FS dehnad A. The impact of synbiotic on serum sCD163/sTWEAK, paraoxonase 1, and lipoproteins in patients with chronic heart failure: a randomized, triple-blind, controlled trial. Sci Rep. (2024) 14:19120. doi: 10.1038/s41598-024-69560-8, 39155305 PMC11330970

[13] Li YL Zhang YZ Zhang YF Dong L Wang WW. Analysis of the impact of oral probiotics on the gut microbiota of patients with chronic heart failure. J Pract Clin Med. (2024) 28:73–7.

[14] Wang J Meng XX Xu RX. The effect of oral probiotics on the immune function and cardiac function of patients with chronic heart failure. Shenzhen J Integr Tradit Chin West Med. (2023) 33:73–5. doi: 10.16458/j.cnki.1007-0893.2023.08.023

[15] Ayodeji A Cristiane M Alex SF Johannes RH Samuel DM Knut TL. Rifaximin or *Saccharomyces boulardii* in heart failure with reduced ejection fraction: results from the randomized GutHeart trial. EBioMedicine. (2021) 70:103511. doi: 10.1016/j.ebiom.2021.103511 34329947 PMC8339250

[16] Pourrajab B Naderi N Janani L Mofid V Hajahmadi M Dehnad A . Comparison of probiotic yogurt and ordinary yogurt consumption on serum Pentraxin3, NT-proBNP, oxLDL, and ApoB100 in patients with chronic heart failure: a randomized, triple-blind, controlled trial. Food Funct. (2020) 11:10000–10. doi: 10.1039/d0fo01014f, 33119010

[17] Matin SS Shidfar F Naderi N Amin A Hosseini-Baharanchi FS Dehnad A. The effect of Synbiotic consumption on serum NTproBNP, hsCRP and blood pressure in patients with chronic heart failure: a randomized, triple-blind, controlled trial. Front Nutr. (2021) 8:822498. doi: 10.3389/fnut.2021.822498, 35498054 PMC9043653

[18] Karim A Muhammad T Shah I Khan J Qaisar R. A multistrain probiotic reduces sarcopenia by modulating Wnt signaling biomarkers in patients with chronic heart failure. J Cardiol. (2022) 80:449–55. doi: 10.1016/j.jjcc.2022.06.006, 35750555

[19] Chen LN Li SH Zhou J Peng Y Xu F. Study on the intervention effects of probiotics on the readmission rate of heart failure patients and the metabolites of oxylucamine in the gut microbiota. Electrocard Circul. (2023) 42:515–9.

[20] Pourrajab B Naderi N Janani L Hajahmadi M Mofid V Dehnad A . The impact of probiotic yogurt versus ordinary yogurt on serum sTWEAK, sCD163, ADMA, LCAT and BUN in patients with chronic heart failure: a randomized, triple-blind, controlled trial. J Sci Food Agric. (2022) 102:6024–35. doi: 10.1002/jsfa.11955, 35460085

[21] Zhang Y Yang X Wu N Zhang XH Gong G Qian XM. Clinical study on the improvement of cardiac function and quality of life in elderly patients with chronic heart failure by probiotic therapy. Chin J Health Care Med. (2017) 19:210–2.

[22] Nurgaziyev M Kozhakhmetov S Jarmukhanov Z Nurgaziyeva A Sergazy S Issilbayeva A . Impact of probiotics and polyphenols on adults with heart failure: a systematic review and meta-analysis. Eur J Med Res. (2025) 30:313. doi: 10.1186/s40001-025-02538-y, 40259417 PMC12010510

[23] Zannad F Rossignol P Iraqi W. Extracellular matrix fibrotic markers in heart failure. Heart Fail Rev. (2010) 15:319–29. doi: 10.1007/s10741-009-9143-0, 19404737

[24] Duprez DA Gross MD Kizer JR Ix JH Hundley WG Jacobs DR Jr. Predictive value of collagen biomarkers for heart failure with and without preserved ejection fraction: MESA (multi-ethnic study of atherosclerosis). J Am Heart Assoc. (2018) 7:e007885. doi: 10.1161/JAHA.117.007885, 29475876 PMC5866330

[25] Parichatikanond W Luangmonkong T Mangmool S Kurose H. Therapeutic targets for the treatment of cardiac fibrosis and Cancer: focusing on TGF-β signaling. Front Cardiovasc Med. (2020) 7:34. doi: 10.3389/fcvm.2020.00034, 32211422 PMC7075814

[26] Hatamnejad MR Medzikovic L Dehghanitafti A Rahman B Vadgama A Eghbali M. Role of gut microbial metabolites in ischemic and non-ischemic heart failure. Int J Mol Sci. (2025) 26:2242. doi: 10.3390/ijms26052242, 40076864 PMC11900495

[27] Kummen M Mayerhofer C Vestad B Mayerhofer CCK Broch K Awoyemi A . Gut microbiota signature in heart failure defined from profiling of 2 independent cohorts. J Am Coll Cardiol. (2018) 71:1184–6. doi: 10.1016/j.jacc.2017.12.057, 29519360

[28] Zhao X Kwan J Yip K Kwan JYY Liu PP Liu FF. Targeting metabolic dysregulation for fibrosis therapy. Nat Rev Drug Discov. (2020) 19:57–75. doi: 10.1038/s41573-019-0040-5, 31548636

[29] Tuerhongjiang G Guo M Qiao X Liu J Xi W Wei Y . Gut microbiota regulate saturated free fatty acid metabolism in heart failure. Small Sci. (2024) 4:2300337. doi: 10.1002/smsc.202300337, 40212081 PMC11935106

[30] Fink MP. Leaky gut hypothesis: a historical perspective. Crit Care Med. (1990) 18:579–80. doi: 10.1097/00003246-199005000-00024, 2328605

[31] Chen L Li S Ai L Zhou J Huang J Xu F . The correlation between heart failure and gut microbiome metabolites. Infect Microbes Dis. (2020) 2:136–43. doi: 10.1097/IM9.0000000000000042, 38630083 PMC7769059

[32] Pasini E Aquilani R Testa C Baiardi P Angioletti S Boschi F . Pathogenic gut flora in patients with chronic heart failure. JACC Heart Fail. (2016) 4:220–7. doi: 10.1016/j.jchf.2015.10.009, 26682791

[33] Brasier AR. The nuclear factor-kappaB-interleukin-6 signalling pathway mediating vascular inflammation. Cardiovasc Res. (2010) 86:211–8. doi: 10.1093/cvr/cvq076, 20202975 PMC2912657

[34] Markousis-Mavrogenis G Tromp J Ouwerkerk W Devalaraja M Anker SD Cleland JG . The clinical significance of interleukin-6 in heart failure: results from the BIOSTAT-CHF study. Eur J Heart Fail. (2019) 21:965–73. doi: 10.1002/ejhf.1482, 31087601

[35] Wang J Wang M Lu X Zhang Y Zeng S Pan X . IL-6 inhibitors effectively reverse post-infarction cardiac injury and ischemic myocardial remodeling via the TGF-β1/Smad3 signaling pathway. Exp Ther Med. (2022) 24:576. doi: 10.3892/etm.2022.11513, 35949328 PMC9353402

[36] Liu Y Sun X Yuan M Yu Z Hou Q Jia Z . Enhanced lipid metabolism reprogramming in CHF rats through IL-6-mediated cardiac glial cell modulation by digilanid C and electroacupuncture stimulation combination. Front Cell Dev Biol. (2024) 12:1424395. doi: 10.3389/fcell.2024.1424395, 39291267 PMC11405320

[37] Schumacher SM Naga Prasad SV. Tumor necrosis factor-α in heart failure: an updated review. Curr Cardiol Rep. (2018) 20:117. doi: 10.1007/s11886-018-1067-7, 30259192 PMC6311126

[38] Zhang QL Chen XH Zhou SJ Lei YQ Chen Q Cao H. Relationship between heart failure and intestinal inflammation in infants with congenital heart disease. BMC Microbiol. (2024) 24:98. doi: 10.1186/s12866-024-03229-0, 38528458 PMC10962087

[39] Wang JL Yang L Shi L. The changes in the intestinal flora of doxorubicin-induced heart failure rats and the effects of probiotics on the expression of NLRP3 and cardiac function in these rats. Chin J Gerontol. (2022) 42:4064–7.

[40] Lucas LN Barrett K Kerby RL Zhang Q Cattaneo LE Stevenson D . Dominant bacterial phyla from the human gut show widespread ability to transform and conjugate bile acids. mSystems. (2021) 6:e0080521. doi: 10.1128/mSystems.00805-21, 34463573 PMC12338150

[41] Tang W Bäckhed F Landmesser U Hazen SL. Intestinal microbiota in cardiovascular health and disease: JACC state-of-the-art review. J Am Coll Cardiol. (2019) 73:2089–105. doi: 10.1016/j.jacc.2019.03.024, 31023434 PMC6518422

[42] Arpaia N Campbell C Fan X Dikiy S van der Veeken J deRoos P . Metabolites produced by commensal bacteria promote peripheral regulatory T-cell generation. Nature. (2013) 504:451–5. doi: 10.1038/nature12726, 24226773 PMC3869884

[43] Marques FZ Nelson E Chu PY Horlock D Fiedler A Ziemann M . High-Fiber diet and acetate supplementation change the gut microbiota and prevent the development of hypertension and heart failure in hypertensive mice. Circulation. (2017) 135:964–77. doi: 10.1161/CIRCULATIONAHA.116.024545, 27927713

[44] Wen J Li L Ou D Li J Yang Y Duan L . Higenamine protects against doxorubicin-induced heart failure by attenuating ferroptosis via modulating the Nrf2/GPX4 signaling pathway. Phytomedicine. (2025) 141:156670. doi: 10.1016/j.phymed.2025.156670, 40220414

[45] Modrego J Ortega-Hernández A Goirigolzarri J Restrepo-Córdoba MA Bäuerl C Cortés-Macías E . Gut microbiota and derived short-chain fatty acids are linked to evolution of heart failure patients. Int J Mol Sci. (2023) 24:13892. doi: 10.3390/ijms241813892, 37762194 PMC10530267

[46] Violi F Castellani V Menichelli D Pignatelli P Pastori D. Gut barrier dysfunction and endotoxemia in heart failure: a dangerous connubium. Am Heart J. (2023) 264:40–8. doi: 10.1016/j.ahj.2023.06.002, 37301317

[47] Huang J Lin Y Ding X Lin S Li X Yan W . Alteration of the gut microbiome in patients with heart failure: a systematic review and meta-analysis. Microb Pathog. (2024) 192:106647. doi: 10.1016/j.micpath.2024.106647, 38788811

[48] Yusof RM Haque F Ismail M Hassan Z. Isolation of *Bifidobacteria infantis* and its antagonistic activity against ETEC 0157 and *Salmonella typhimurium* S-285 in weaning foods. Asia Pac J Clin Nutr. (2000) 9:130–5. doi: 10.1046/j.1440-6047.2000.00154.x, 24394399

[49] Ruas-Madiedo P Gueimonde M Margolles A de Los Reyes-Gavilán CG Salminen S. Exopolysaccharides produced by probiotic strains modify the adhesion of probiotics and enteropathogens to human intestinal mucus. J Food Prot. (2006) 69:2011–5. doi: 10.4315/0362-028x-69.8.2011, 16924934

[50] Groeger D O'Mahony L Murphy EF Bourke JF Dinan TG Kiely B . *Bifidobacterium infantis* 35624 modulates host inflammatory processes beyond the gut. Gut Microbes. (2013) 4:325–39. doi: 10.4161/gmic.25487, 23842110 PMC3744517

[51] Hayashi T Yamashita T Watanabe H Kami K Yoshida N Tabata T . Gut microbiome and plasma microbiome-related metabolites in patients with decompensated and compensated heart failure. Circ J. (2018) 83:182–92. doi: 10.1253/circj.CJ-18-0468, 30487369

[52] Lv S Wang Y Zhang W Shang H. Trimethylamine oxide: a potential target for heart failure therapy. Heart. (2022) 108:917–22. doi: 10.1136/heartjnl-2021-320054, 34611047

[53] Cantero MA Guedes M Fernandes R Lollo P. Trimethylamine N-oxide reduction is related to probiotic strain specificity: a systematic review. Nutr Res. (2022) 104:29–35. doi: 10.1016/j.nutres.2022.04.001, 35588611

