# Supplementary material for: Effects of probiotics on heart failure: a systematic review and meta-analysis
Source: Front Nutr. 2025 Dec 5;12:1708678. doi: 10.3389/fnut.2025.1708678 (PMC12755241; doi:10.3389/fnut.2025.1708678)
Supplement: Supplementary file 1 [file Table_1.docx]

| **1.Pubmed n=316** | |
| --- | --- |
| Search number | Query |
| #1 | ("Heart Failure"[Mesh]) OR ((((((((((((((Cardiac Failure[Title/Abstract]) OR (Heart Decompensation[Title/Abstract])) OR (Decompensation, Heart[Title/Abstract])) OR (Congestive Heart Failure[Title/Abstract])) OR (Heart Failure, Congestive[Title/Abstract])) OR (Heart Failure, Right-Sided[Title/Abstract])) OR (Heart Failure, Right Sided[Title/Abstract])) OR (Right-Sided Heart Failure[Title/Abstract])) OR (Right Sided Heart Failure[Title/Abstract])) OR (Heart Failure, Left-Sided[Title/Abstract])) OR (Heart Failure, Left Sided[Title/Abstract])) OR (Left-Sided Heart Failure[Title/Abstract])) OR (Left Sided Heart Failure[Title/Abstract])) OR (Myocardial Failure[Title/Abstract])) |
| #2 | ((((("Probiotics"[Mesh]) OR ((Probiotic[Title/Abstract]))) OR (("Synbiotics"[Mesh]) OR (Synbiotic[Title/Abstract]))) OR (("Yeasts"[Mesh]) OR (Yeast[Title/Abstract]))) OR (("Lactobacillales"[Mesh]) OR (Lactic Acid Bacteria[Title/Abstract]))) OR ("Bifidobacterium"[Mesh]) |
| #3 | #1 AND #2 |

1.Search strategy

| **2.Cochrane n=31** | |
| --- | --- |
| Search number | Query |
| #1 | MeSH descriptor: [Heart Failure] explode all trees |
| #2 | (Cardiac Failure):ti,ab,kw OR (Heart Decompensation):ti,ab,kw OR (Decompensation, Heart):ti,ab,kw OR (Congestive Heart Failure):ti,ab,kw OR (Heart Failure, Congestive):ti,ab,kw OR (Heart Failure, Right-Sided):ti,ab,kw OR (Heart Failure, Right Sided):ti,ab,kw OR (Right-Sided Heart Failure):ti,ab,kw OR (Right Sided Heart Failure):ti,ab,kw OR (Heart Failure, Left-Sided):ti,ab,kw OR (Heart Failure, Left Sided):ti,ab,kw OR (Left-Sided Heart Failure):ti,ab,kw OR (Left Sided Heart Failure):ti,ab,kw OR (Myocardial Failure):ti,ab,kw |
| #3 | #1 OR #2 |
| #4 | MeSH descriptor: [Probiotics] explode all trees |
| #5 | (Probiotic):ti,ab,kw |
| #6 | MeSH descriptor: [Synbiotics] explode all trees |
| #7 | (Synbiotic):ti,ab,kw |
| #8 | MeSH descriptor: [Yeasts] explode all trees |
| #9 | (Yeast):ti,ab,kw |
| #10 | MeSH descriptor: [Bifidobacterium] explode all trees |
| #11 | MeSH descriptor: [Lactobacillales] explode all trees |
| #12 | (Lactic Acid Bacteria):ti,ab,kw |
| #12 | #4 OR #5 OR #6 OR #7 OR #8 OR #9 OR #10 OR #11 OR #12 |
| #14 | #3 AND #13 |

| **3.Embase n=4308** | |
| --- | --- |
| Search number | Query |
| #1 | 'heart failure'/exp |
| #2 | 'heart failure':ab,ti OR 'cardiac failure':ab,ti OR 'heart decompensation':ab,ti OR 'decompensation, heart':ab,ti OR 'congestive heart failure':ab,ti OR 'heart failure, congestive':ab,ti OR 'heart failure, right-sided':ab,ti OR 'heart failure, right sided':ab,ti OR 'right-sided heart failure':ab,ti OR 'right sided heart failure':ab,ti OR 'heart failure, left-sided':ab,ti OR 'heart failure, left sided':ab,ti OR 'left-sided heart failure':ab,ti OR 'left sided heart failure':ab,ti OR 'myocardial failure':ab,ti |
| #3 | #1 OR #2 |
| #4 | 'probiotic agent'/exp OR 'synbiotic agent'/exp OR 'yeast'/exp OR 'bifidobacterium'/exp OR 'lactobacillales'/exp |
| #5 | 'probiotic agent':ab,ti OR 'probiotic':ab,ti OR 'synbiotic agent':ab,ti OR 'synbiotic':ab,ti OR 'bifidobacterium':ab,ti OR 'yeast':ab,ti OR 'lactobacillales':ab,ti OR 'lactic acid bacteria':ab,ti |
| #6 | #4 OR #5 |
| #7 | #3 AND #6 |

| **4.Web of science n=294** | |
| --- | --- |
| Search number | Query |
| #1 | ((((((((((((((TS=(Heart Failure)) OR TS=(Cardiac Failure)) OR TS=(Heart Decompensation)) OR TS=(Decompensation, Heart)) OR TS=(Congestive Heart Failure)) OR TS=(Heart Failure, Congestive)) OR TS=(Heart Failure, Right-Sided)) OR TS=(Heart Failure, Right Sided)) OR TS=(Right-Sided Heart Failure)) OR TS=(Right Sided Heart Failure)) OR TS=(Heart Failure, Left-Sided)) OR TS=(Heart Failure, Left Sided)) OR TS=(Left-Sided Heart Failure)) OR TS=(Left Sided Heart Failure)) OR TS=(Myocardial Failure) and Preprint Citation Index (Exclude – Database) |
| #2 | ((((((((((TS=(Probiotics)) OR TS=(Probiotic)) OR TS=(Synbiotics)) OR TS=(Synbiotic)) OR TS=(Yeasts)) OR TS=(Yeast)) OR TS=(Bifidobacterium)) OR TS=(Bacillus)) OR TS=(Bacillus bacterium)) OR TS=(Lactobacillales)) OR TS=(Lactic Acid Bacteria) and Preprint Citation Index (Exclude – Database) |
| #3 | #1 AND #2 |
|  |  |

| **5.CNKI n=23** | |
| --- | --- |
| Search number | Query |
| #1 | (SU=(心力衰竭) AND SU=(益生菌 OR合生元 OR 酵母菌 OR 双歧杆菌 OR 乳酸菌)) |

| **6.Wanfang Database n=0** | |
| --- | --- |
| Search number | Query |
| #1 | 主题:(益生菌OR合生元OR酵母菌OR双歧杆菌OR芽孢杆菌OR乳酸菌) and 主题:(心力衰竭) |

| **7.the Chinese Clinical Trial Registry n=0** | |
| --- | --- |
| Search number | Query |
| #1 | In the "研究疾病名称" box, enter "心力衰竭". In the "干预措施" box, respectively input "益生菌、合生元、酵母菌、双歧杆菌、芽孢杆菌、乳酸菌". |

| **8.ClinicalTrials.gov n=0** | |
| --- | --- |
| Search number | Query |
| #1 | In the "Condition/disease" search box, enter "Heart failure". In the "Intervention/treatment" search box, respectively input "Probiotics, Synbiotics, Yeasts, Bifidobacterium, Bacillus, Lactobacillales". |

1. Consistency test of literature screening and quality evaluation

|  |  | Researcher 2 | |  |
| --- | --- | --- | --- | --- |
|  |  | Inclusion | Exclude | Total |
| Researcher 1 | Inclusion | 11 | 2 | 13 |
|  | Exclude | 0 | 4959 | 4959 |
|  | Total | 11 | 4961 | 4972 |
| Kappa test | Kappa=0.916 | *P* < 0.05 |  |  |

|  |  | Researcher 2 | | |  |
| --- | --- | --- | --- | --- | --- |
|  |  | High quality | Medium quality | Low quality | Total |
| Researcher 1 | High quality | 4 | 0 | 0 | 4 |
|  | Medium quality | 1 | 1 | 1 | 3 |
|  | Low quality | 0 | 0 | 4 | 4 |
|  | Total | 5 | 1 | 5 | 11 |
| Kappa test | Kappa = 0.718 | *P* < 0.05 |  |  |  |

During the literature screening stage, Kappa = 0.916, P < 0.05; in the quality evaluation stage, Kappa = 0.718, P < 0.05. Both stages achieved a strong consistency, indicating that the screening and evaluation results were reliable.
