# Supplementary figures and images for: Effects of probiotics on heart failure: a systematic review and meta-analysis
Source: Front Nutr. 2025 Dec 5;12:1708678. doi: 10.3389/fnut.2025.1708678 (PMC12755241; doi:10.3389/fnut.2025.1708678)

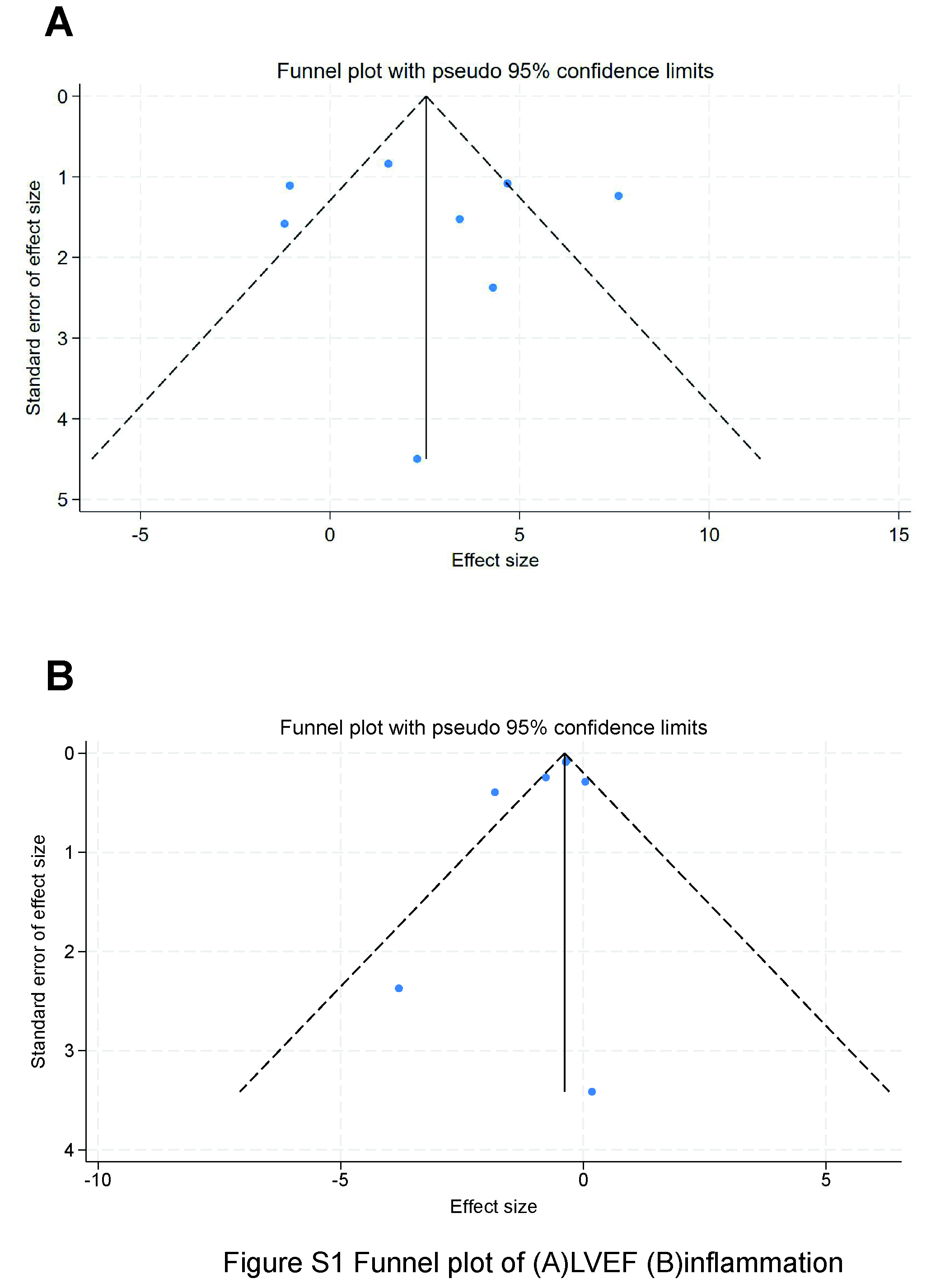

Supplement: Supplementary file 2 [file Image_1.tif]
